# Imposter Participants in Online Nursing Research: Prevalence, Red Flags, and Risk Mitigation Strategies

**DOI:** 10.3390/nursrep16040122

**Published:** 2026-04-03

**Authors:** Richard J. Gray, Niall Higgins, Piyanee Yobas, Alessandro Stievano, Daniel Bressington

**Affiliations:** 1 School of Nursing and Midwifery, La Trobe University, Melbourne, VIC 3096, Australia; 2 School of Nursing, Midwifery and Social Work, University of Southern Queensland, Toowoomba, QLD 4350, Australia; niall.higgins@health.qld.gov.au; 3 Alice Lee Centre for Nursing Studies, National University of Singapore, Singapore 117599, Singapore; nurpk@nus.edu.sg; 4 Centre of Excellence for Nursing Scholarship, International Medical University in Rome, 00131 Rome, Italy; alessandro.stievano@gmail.com; 5 Faculty of Nursing, Chiang Mai University, Chiang Mai 50200, Thailand; daniel.bressington@cmu.ac.th

## 1. Definitions, Prevalence, and Red Flags

Following the COVID-19 pandemic, there has been substantial growth in the amount of nursing research conducted online, with participants often recruited via social media platforms [1]. Whilst there are potential benefits to online research, such as increased reach and more representative samples, there are important risks, most notably the recruitment of imposter participants [2,3]. As nursing research plays an expanding role in shaping clinical guidelines, workforce policy, and models of care, imposter participation undermines scientific validity and poses downstream risks to patient safety and service planning. This concern is increasingly being recognised across health research as a growing threat to data integrity, particularly in studies relying on online recruitment and data collection [4].

Imposter participants (also referred to as fraudulent, suspicious, or ineligible participants) provide false or misleading information about their eligibility to gain access to a study [2,5]. There are two main kinds of imposter participants: 1. people who provide false or deceptive responses (e.g., professional status, diagnosis, or lived experience) and 2. automated or bot-generated responses designed to simulate legitimate participant engagement, particularly in survey-based studies. It is important to understand, though, that imposter participation should be distinguished from inadvertent ineligibility or ambiguous self-identification, as these represent fundamentally different ethical and methodological challenges.

A systematic review, involving 15 studies, reported that imposter participants were observed across countries (including Australia, Canada, UK, and USA), disciplines (including nursing), and populations (including healthcare providers, caregivers, young people, marginalised groups, and stigmatised populations) [6]. Researchers across the included studies conceptualised the imposter participant issue as “deceiving individuals”, “deceptive behaviours”, and “purposeful misrepresentation”, among others. The impact of imposter participants was categorised into three philosophical matters: 1. threats to the accessibility and diversity of populations, 2. trust and ethics, and 3. the integrity of research findings. Furthermore, authors of all 15 studies indicated that financial incentive was a primary driver for imposter behaviour [6]. A clear potential motivation for imposter participants is financial gain [7]. Many researchers offer payment to participants for completing online surveys or taking part in qualitative interviews conducted using video communication platforms such as MS TEAMS or ZOOM. Generally, the size of the payment is modest, for example, a $10 or $20 gift card for completing an online survey; however, some lived experience patient groups have advocated that participants should be paid substantially higher amounts for taking part in research to more legitimately reflect their contribution to the work [8]. We have identified studies where participants are paid up to $100 (AUD) for participating in interviews [9,10], so it is easy to see how participation in research can become a lucrative source of income for individuals or organised groups. Whilst online research recruitment platforms are attractive for some researchers as being convenient and timesaving, the main messages they seem to convey to potential research participants is one of monetary gain, with little focus on altruistic motivation. Examples of straplines on some of the most popular recruitment websites include: “Start earning money today by sharing your opinions”, “Find paid remote and in-person research studies you’ll be well-compensated”, “Cash in on Curiosity: How to Participate in Paid Studies Online”.

Money might be the most common reason, but it is not the only motivation for imposter participants. There are relevant case studies in the literature where imposters have signed up for a study with the deliberate intent of sabotaging the research [5,11]. For example, in a study on LGBTQ+ parents, authors observed that imposter participants were not meaningfully completing the survey, criticised the gender identity question, and left offensive comments in the free-text section of the survey [11].

The scale of the problem is difficult to establish; we have not been able to locate studies that have estimated the prevalence of imposter participants in online research in any discipline. Methodologically, such a study would be challenging to conduct, as seemingly many researchers are unaware that their studies may have involved fraudulent participants. One scoping review included 23 studies where authors explicitly described strategies to detect and address imposter participants during the online recruitment process [12]. The authors of 18 of the 23 (78%) included studies identified imposter participants had been included. Between 3% and 94% of participants in the sample were considered fraudulent [12]. This review provides some, albeit limited, evidence suggesting that imposter participants may be extremely prevalent in online research where people taking part are paid.

One mixed-methods study sought to explore healthcare researchers’ experience of imposter participants [7]. The study sought to develop an understanding as to how researchers identified and mitigated against imposter participants in their research. Thirty-seven researchers took part in the study, mostly recruiting via social media for qualitative or mixed-methods research, with a survey being the most common approach to data collection. The authors described how researchers found it ‘scary’ and ‘surprising’ when they discovered that they had enrolled fraudulent participants to their study. One researcher-participant reported that they found it ‘astonishing’ that people would invest time enrolling in a study with a prize draw where there was no guarantee they would win. Researcher suspicion was triggered by a high volume of responses to surveys at the same time, a lack of contextual information (e.g., how participants learned about the study) in email responses offering to take part in research, or the use of stereotypical Western names (e.g., John Smith) or famous people (e.g., Taylor Swift).

There are numerous case studies in the literature where researchers describe their experiences of being ‘scammed’ by imposter participants that we encourage colleagues to read (see for example, [9,13]). From reviewing these case studies and from talking to researchers who have had to deal with imposter participants, several ‘Red Flags’ [14] emerge that colleagues conducting online research should pay close attention to:

During recruitment:

1. Larger-than-anticipated responses to the survey or expressions of interest in participating in research submitted within a short time frame (typically a few hours), often at unexpected times of the day (e.g., overnight).
2. Emails from different potential participants sent to researchers that are identical or extremely similar.
3. Curious email addresses that use random letters or old-fashioned names.
4. Short emails to the researcher that are vague or extremely short (e.g., ‘I’m interested’ or ‘Send me the link’).
5. Multiple emails or survey responses from the same IP (internet protocol) address.
6. Emails that do not contain a subject line.
7. Emails with short and fragmented sentences that often contain immediate queries about participant incentives.
8. Signing a consent form with a date that is not in a correct format for a specific country (such as using day/month/year for a study conducted in the USA).

Participant characteristics:

1. During interviews, participants claim to be working in a particular region but are unable to provide simple contextual detail (e.g., claim to work in rural Australia but cannot name the town or city in which they are working).
2. Implausibly high numbers of potential participants from specific groups, for example, people who identify as being from an Indigenous background.
3. Discrepancies between participant-reported and system-captured data (e.g., location, time zone).

Conduct during interviews:

1. Declining to turn on their video camera during interviews without reasonable justification.
2. Vague, brief, or seemingly scripted responses to interview questions that lack the expected level of detail.
3. Excessive focus on payment (e.g., ‘when will I get paid’, ‘how long will it take to get my voucher’).
4. Repeating or parroting the responses of other respondents in a focus group interview.

Responses to surveys:

1. Surveys are completed unexpectedly quickly.
2. Multiple survey responses are identical or near identical.
3. Hidden survey items are completed.

Post-participation conduct:

1. Efforts to claim multiple payments for participating (e.g., by claiming voucher codes were incorrect).
2. Failure to respond to follow-up emails (e.g., asking participants to check interview transcripts).

Each ‘tell’ is not definitive proof of fraudulent participation but should serve as a prompt to researchers that they should investigate further.

## 2. Impact

It is an old adage—garbage in, garbage out; imposter participants often contribute inconsistent, poor-quality responses and are consequently a serious threat to research integrity in many disciplines, including nursing. Only a minority of researchers have likely recognised they have included imposter participants, and it is reasonable to deduce that there are many published studies that include fraudulent participants. Once a paper is published, it is still possible to detect imposter participants; for example, data can be rechecked for anomalies after publication. But we can find no examples of studies being retracted for this reason. This possibly suggests that retraction, for this type of scientific ‘error’, may not yet be a mechanism that is currently working for correcting this integrity issue.

## 3. Addressing the Problem

Responsibility for addressing the issue of imposter participants, at least in part, lies with researchers who need to be aware and vigilant. Methodological guidance has also highlighted the ethical trade-offs inherent in participant verification, particularly the need to balance fraud prevention with respect for privacy and inclusivity [15]. Researchers planning to conduct online research should include a detailed risk mitigation plan as part of their study protocol. Risk of imposter participation spans the full research life cycle, from study design and recruitment through to data collection, analysis, and reporting and cannot be addressed through recruitment screening alone.

When developing an imposter participant risk mitigation strategy, we suggest researchers consider the following:

Recruitment:

1. Avoid open social media platforms; if possible, use trusted or closed social media groups.
2. Request evidence of professional registration if recruiting nurses of other healthcare professionals.
3. Seek details from participants of their workplace (e.g., name of hospital) and location (e.g., town, city) that can be verified.
4. Check IP (internet protocol) addresses to confirm location.
5. Conduct a brief online screening interview requesting participants turn on their camera, noting sensitivity to possible privacy concerns.
6. Consider conducting the recruitment in two stages—an initial screening interview to assess eligibility criteria, followed by the main survey. The main survey could then include some duplicated eligibility questions, and any inconsistencies can be investigated [16].

Interview and data collection techniques:

1. Design interviews to elicit details of relevant lived experience.
2. At the start of the interview, ask specific questions relating to study eligibility criteria.
3. Attend to vague, inconsistent, or possibly scripted responses to interview questions.
4. Document unusual interview conduct that might include delayed responses or refusal to turn on their camera.
5. Document in a field notes researcher impressions of participant authenticity and engagement.

Payment to participants:

1. Carefully consider the level of participant payment.
2. Use a gift card that can only be used in relevant countries.
3. Clearly state in participant information documentation that payment will only be made following data checking.
4. Consider a prize draw. Guaranteed incentives have been associated with better response rates but also higher fraudulent responses [16]. Framing compensation as being based on chance rather than a payment, i.e., a lottery, can reduce the number of bot responses as the underpinning AI will aim to avoid participation where payment is not guaranteed [17].

Technical and analytical strategies:

1. Where possible, use CAPTCHA (completely automated public Turing test to tell computers and humans apart) to authentic participants are human.
2. Use online survey software that can collect metadata, including time spent on completing an online survey (i.e., not too fast, not to slow, not from different locations).
3. Monitor duplicate IP (internet protocol) addresses and metadata anomalies.
4. Check demographic data with publicly available population characteristics.

Despite growing recognition of the problem, there remains little consensus on how researchers should ethically manage data generated by suspected imposter participants once collected, including whether such data should be retained, excluded, or transparently reported [1,3,14,15].

Given all the recommended safeguard measures, some imposter participants may manage to enter the study and attend all research-related activities. It is suggested that suspicious participants be compensated for their participation [14]. However, researchers should pay close attention during the data analysis phase. Researchers should consider: 1. criteria to determine potential imposters, 2. strategies to manage such imposters (such as removal of all data or some data), and 3. how to report questionable data in the (limitations section) of the manuscript [14].

An imposter participant protocol was presented in a qualitative study on LGBTQ individuals, comprising six stages [18]. Researchers in this study were required to ask certain questions and perform activities for each phase. The conceptualise stage began before the commencement of the study. As an example, members of the target population were invited to serve as a co-investigator or advisory board member to ascertain participant identification. The recruitment stage unitised slow recruitment with constant validation to ensure participants were genuine. The screening stage involved a pilot test of screening questions on members of the target population. This aimed to obtain ideas of how they might respond. During the data collection stage, researchers observed and noted the presence of certain behaviours (such as being in a noisy space during the qualitative interview and giving brief answers that require significant probing questions). The analysis stage used a collective reflective journal, addressing issues of potential imposter participants, team discussion, and researchers’ judgement on the issue [18]. Finally, the reporting stage involved discussions about the process of determining and managing the imposter issues. Such experiences were used to inform future research, especially in qualitative studies [18].

The responsibility of addressing the challenge posed by imposter participants does not sit solely with researchers; universities, research institutions, and health services also have an important role to play. Institutions should consider:

1. Developing institutional guidance and training on managing imposter participants in human research.
2. Asking researchers to provide a risk mitigation plan as part of the human ethics application process.
3. Encouraging the reporting of fraud prevention strategies in publications including details of any imposter participants that were identified and excluded.
4. Encouraging community involvement and engagement for the recruitment and data collection plan. Community engagement with a direct link to target participants, will help identify genuine participants.

## 4. Reporting Guidelines

Most researchers will draft their research manuscript against the relevant reporting guideline (e.g., COREQ [19], STROBE [20]) to ensure a comprehensive and transparent reporting of the work. Although some reporting guidelines are reasonably frequently updated, as best as we can determine, they do not currently require authors to describe risk mitigation against the inclusion of fraudulent participants or report on the number included in the study and if they were excluded from the results. This limitation persists despite detailed methodological guidance in, for example, the STROBE explanation and elaboration documents [21]. At a minimum, reporting guidelines should require authors to disclose the use of online recruitment, describe participant verification procedures, and report the number of suspected imposter participants identified and excluded. We consider that there is a pressing need for the authors of reporting guidelines to update them to include relevant items on imposter participants. This seems particularly pressing for guidelines that relate to qualitative (e.g., COREQ) and observational (e.g., STROBE) research.

## 5. Editorial Position

As an editorial team, we consider that we also have a role to play in addressing the threat imposter participants pose to the integrity of the research that we publish. In this editorial, we seek to raise awareness among nurse researchers about this issue. We also consider that there are additional strategies that we can implement to support researchers and research trainees.

1. We will publish a journal policy statement as part of our author guidelines on participant verification and fraud prevention in research.
2. Authors will be asked to declare (at the end of their manuscript) if any study participants were recruited using online platforms. If participants were recruited online, authors will then need to describe (in the body of the paper) the risk mitigation strategies that were in place to ensure the authenticity of people enrolled in the research.
3. For studies that used online recruitment, authors will need to include in the manuscript details of the number of imposter participants identified and if they were excluded from the study.
4. The value of open data sharing will be emphasised for authors submitting to *Nursing Reports.* Researchers will also be encouraged to alert us if they suspect, post-publication, that their paper included imposter participants.
5. We will provide additional training and guidance to reviewers about imposter participants and how they should address the issue when drafting review reports.

## 6. Conclusions

Imposter participants are a real and arguably existential threat to the integrity of nursing research. We acknowledge that the empirical evidence base remains emergent and that the scale and characteristics of imposter participation are likely to vary across disciplines, populations, and study designs. Nurse researchers need to be proactive in addressing this risk when developing their study protocol and ensure that they have a clear mitigation strategy in place. There is also work that needs to take place at an institutional level to educate and train researchers. As an editorial team at *Nursing Reports*, we take this issue seriously and aim to support authors and reviewers in ensuring the research we publish is both of high quality and impactful.

## Data Availability

Not applicable.
